# Feasibility of in vivo multi-parametric quantitative magnetic resonance imaging of the healthy sciatic nerve with a unified signal readout protocol

**DOI:** 10.1038/s41598-023-33618-w

**Published:** 2023-04-21

**Authors:** Ratthaporn Boonsuth, Marco Battiston, Francesco Grussu, Christina Maria Samlidou, Alberto Calvi, Rebecca S. Samson, Claudia A. M. Gandini Wheeler-Kingshott, Marios C. Yiannakas

**Affiliations:** 1 NMR Research Unit, Department of Neuroinflammation, Faculty of Brain Sciences, Queen Square MS Centre, UCL Queen Square Institute of Neurology, University College London, London, UK; 2 Department of Radiologic Technology, Faculty of Associated Medical Sciences, Chiang Mai University, Chiang Mai, Thailand; 3 Laboratory of Advanced Imaging in Neuroimmunological Diseases, Center of Neuroimmunology, Hospital Clinic Barcelona, Fundació Clinic Per a La Recerca Biomedica, Barcelona, Spain; 4 Brain Connectivity Research Centre, IRCCS Mondino Foundation, Pavia, Italy; 5 Department of Brain and Behavioural Sciences, University of Pavia, Pavia, Italy

**Keywords:** Neurology, Peripheral nervous system

## Abstract

Magnetic resonance neurography (MRN) has been used successfully over the years to investigate the peripheral nervous system (PNS) because it allows early detection and precise localisation of neural tissue damage. However, studies demonstrating the feasibility of combining MRN with multi-parametric quantitative magnetic resonance imaging (qMRI) methods, which provide more specific information related to nerve tissue composition and microstructural organisation, can be invaluable. The translation of emerging qMRI methods previously validated in the central nervous system to the PNS offers real potential to characterise in patients in vivo the underlying pathophysiological mechanisms involved in a plethora of conditions of the PNS. The aim of this study was to assess the feasibility of combining MRN with qMRI to measure diffusion, magnetisation transfer and relaxation properties of the healthy sciatic nerve in vivo using a unified signal readout protocol. The reproducibility of the multi-parametric qMRI protocol as well as normative qMRI measures in the healthy sciatic nerve are reported. The findings presented herein pave the way to the practical implementation of joint MRN-qMRI in future studies of pathological conditions affecting the PNS.

## Introduction

Magnetic resonance imaging (MRI), in particular magnetic resonance neurography (MRN), has been used extensively over the years to investigate the peripheral nervous system (PNS), providing early identification and exact localisation of neural tissue damage with high sensitivity. Indeed, MRN has been demonstrated to identify PNS damage in inflammatory, neoplastic, metabolic and traumatic pathologic conditions^1–3^. More recently, MRN has also been used successfully in combination with other MRI methods both in animal models and in vivo to obtain more specific information regarding the nerve tissue composition, thus enabling better understanding of the underlying pathophysiological mechanisms involved^4,5^. However, the feasibility of combining MRN with more advanced quantitative MRI (qMRI) acquisitions such as multi-shell diffusion-weighted imaging (DWI), quantitative magnetisation transfer (qMT) and T1 relaxometry, known to characterise alterations in tissue composition and microstructure in the presence of pathology^6^, has not been thoroughly explored in vivo.

DWI is sensitive to the ubiquitous self-diffusion of water molecules in neural tissue and enables the inference of tissue organisation at the microscopic level (i.e., of microstructure) within each imaging voxel^7^. Among several DWI methods, diffusion tensor imaging (DTI)^8^, relies on the fitting of a mono-exponential signal representation of Gaussian diffusion to sets of diffusion-weighted signals, which are typically acquired at fixed, intermediate diffusion weighting (b-value) and for a number of uniformly distributed gradient directions. This enables mapping the 6 independent components of the diffusion tensor, which can be combined to reconstruct metrics characterising the three-dimensional displacement profile of water molecules due to diffusion, e.g., axial, radial, mean diffusivity (AD, RD, MD, respectively) and fractional anisotropy (FA). DTI has been used extensively over the years to study the healthy PNS at various anatomical locations demonstrating normal variation in DTI metrics^9–11^, and in various pathological conditions where it was able to characterise axon and myelin integrity^12–16^.

A new series of models or signal representations leveraging multiple b-values (i.e., multi-shell) have also been developed recently, expanding the options of characterising neural tissue in vivo. Measurements at multiple, normally higher b-values, allows the study of more specific aspects of the cellular environment^17^. Diffusion kurtosis imaging (DKI) is one such multi-shell DWI approach^18^. DKI extends DTI naturally by including an additional tensor to the signal representation (the kurtosis tensor), which characterises the non-Gaussian properties of water diffusion within a voxel with the aim of increasing the sensitivity to microstructural characteristics, such as presence of diffusion restrictions, reflected in the reconstructed metrics e.g., axial, radial, mean kurtosis (AK, RK, MK, respectively). However, in spite of its promises, DKI applications in the context of PNS assessment remain largely unexplored, even though it offers great promise for assessing peripheral nerve microstructural features, both in health and in disease state.

The ‘magnetisation transfer (MT) effect’ refers to the transfer of saturation via chemical exchange and dipole–dipole interactions between the ‘free’ water protons (such as those in free water molecules) and the ‘bound’ protons (such as those found in the macromolecules). Generally, the MT effect is created by means of dedicated preparation schemes that selectively saturate macromolecules magnetisation, exploiting their short transverse relaxation time and broad spectrum of resonance frequencies^19,20^. The MT effect is typically measured with the magnetisation transfer ratio (MTR)^21^, obtained by intensity normalisation of an MT-weighted image with a non-saturated one, and has been shown to correlate well with tissue myelin content in the central nervous system (CNS)^22,23^. MTR has been used to study the healthy PNS at various anatomical locations reporting normative MTR values^24–26^, and in the presence of pathology, demonstrating a reduction in MTR values suggestive of demyelination^27,28^^.^

Various qMT imaging techniques have gradually been introduced over the years, to overcome the intrinsic limitations of the MTR, such as its semi-quantitative nature and its dependence on sequence design specifications. These techniques are typically based on a two-pool description of biological tissues (i.e., free pool F and bound pool B), require knowledge of the observed longitudinal relaxation rate (T1_obs_), and rely on fitting an appropriate model of the acquired signal to a series of MT-weighted images in order to separate contributions from the exchange rate from F to B (*k*_FB_), each pool’s transverse relaxation time T2 (i.e., T2_F_ and T2_B_), and the relative size of the bound pool fraction (BPF)^29–31^. These metrics have enabled improved assessment of myelin content as demonstrated in animal studies^32,33^, human in vivo healthy volunteer studies^34,35^, and in the presence of pathology^36,37^, although their potential role in the context of PNS imaging is yet to be determined.

The longitudinal relaxation time T1 is a fundamental quantitative parameter in MRI, which has found a widespread use in clinical studies^38^. In the CNS, T1 relaxation time is mostly dependent on myelin content as it reflects the protons bound to macromolecules^39^, although iron is also known to contribute to these measurements to a certain extent^40^. Due to the quantitative nature of T1 measurements, the confounding effects of sequence-related variations in the signal intensity in T1-weighted images are removed, thus enabling comparison across subjects, scanners and time to enable characterisation of tissue composition. Importantly, accurate knowledge of T1 relaxation time serves as the basis for other quantitative measurements, such as qMT. The role of T1 relaxometry in the study of pathological conditions affecting the PNS is currently unknown, although the technical challenges associated with obtaining accurate T1 relaxation time measurements need to be adequately addressed^41^.

The aim of this pilot study was to assess the feasibility of combining MRN with a uniquely rich multi-parametric qMRI protocol encompassing DKI, qMT and T1 measurements in the healthy sciatic nerve in vivo, by taking into account a number of technical challenges associated with imaging the PNS, and by assessing the reproducibility and reliability of these qMRI measurements.

## Materials and methods

### Study participants

Twelve healthy volunteers (6 male and 6 female, mean age 32.2 years, range 25–40 years) were recruited for this study, all without a history of neuropathy, extremities pain, hypoesthesia or paraesthesia, diabetes mellitus, alcoholism, or any other risk factor for polyneuropathy. All experiments in this research were performed in accordance with the International Conference on Harmonisation Good Clinical practice (ICH GCP) and was approved by the London Harrow Research Ethics Committee (05/Q0502/101). Written informed consent was obtained from all study participants. Half the number of participants (N = 6/12) were called back for a rescan session, as part of the reproducibility assessment.

### MRI acquisition

MR imaging was performed at a single imaging centre using a Philips Ingenia CX 3T MRI system (Philips Medical Systems, Best, Netherlands) with the product SENSE spine and torso coils. In all participants, the right sciatic nerve was assessed. The potential for motion during scanning was minimised using appropriate immobilisation technique involving sandbags and velcro safety straps around the legs, while ensuring that the participants were in a comfortable position; the participants were advanced 'feet-first' into the scanner, and the entire imaging protocol combining MRN with qMRI was acquired in a single session of ~ 55 min.

#### Anatomical imaging—MRN

For visualisation of the sciatic nerves, the 3D ‘nerve-SHeath signal increased with INKed rest-tissue RARE Imaging’ (SHINKEI) sequence was used in the coronal plane with a large field-of-view (FOV)^42^. This image was then used to facilitate prescription of a high-resolution 2D fat-suppressed T2-weighted fast spin-echo (FSE) acquisition in the axial plane, perpendicular to the nerve's longitudinal axis. In all cases, a 12 cm cross-section of the right sciatic nerve (i.e., upper thigh) was assessed, ensuring consistency in the between-subject slice prescription by measuring the distance between the greater trochanter and the lateral femoral condyles using the scanner’s own measuring tools. The imaging parameters were as follows:

- 3D SHINKEI sequence with repetition time (TR) = 2200 ms; echo-time (TE) = 180 ms; FOV = 300 × 420 mm^2^; voxel size = 1.2 × 1.2 × 2 mm^3^; number of excitations (NEX) = 1; turbo spin-echo (TSE) factor = 56; improved motion sensitised driven-equilibrium (iMSDE) duration = 50 ms; 170 slices; scanning time of 5:43 min.
- 2D fat-suppressed T2-weighted FSE acquisition with TR = 5000 ms; TE = 60 ms; FOV = 180 × 180 mm^2^; voxel size = 0.5 × 0.5 × 4 mm^3^; NEX = 1; TSE factor = 11; 30 slices; scanning time of 8:08 min.

#### Quantitative imaging—qMRI

The qMRI protocol consisted of diffusion- and magnetisation transfer-weighted imaging, as well as inversion recovery (IR) for T1 measurement. The protocol was performed using zonally oblique-magnified multislice (ZOOM) EPI^43,44^, in separate acquisitions employing a unified readout approach (i.e., same resolution and FOV)^45^, and with identical scan geometry to the high-resolution fat-suppressed T2-weighted acquisition, using the following parameters:

- Multi-shell DWI with TE = 80 ms; TR = 4000 ms; FOV = 64 × 40 mm^2^; voxel size = 1 × 1 × 10 mm^3^; 12 slices; b-value = 700 s/mm^2^ (16 directions); b-value = 1200 s/mm^2^ (20 directions); b-value = 2000s/mm^2^ (32 directions); 7 interleaved non-diffusion-weighted (b = 0) images were also collected; the scanning time was 16:36 min.
- qMT with TE = 51 ms; TR = 7377 ms; FOV = 64 × 40 mm^2^; voxel size = 1 × 1 × 10 mm^3^; 12 slices; 10 MT weighted images and scanning time of 9:13 min. The B1 map was calculated using the double angle method (DAM)^46^, from two ZOOM EPI volumes acquired with the same resolution and FOV as the quantitative protocol, with TR = 10000 ms, TE = 51 ms, and excitation flip angle of 60 and 120 degrees (scanning time = 1:30 min per volume).
- T1 relaxometry with IR^47^, with TE = 51 ms; TR = 8335 ms; FOV = 64 × 40 mm^2^; voxel size = 1 × 1 × 10 mm^3^; 12 slices; 8 inversion times from 30 to 2130 ms; scanning time of 7:05 min.

### MRI processing

#### Image segmentation

**Figure 1:**
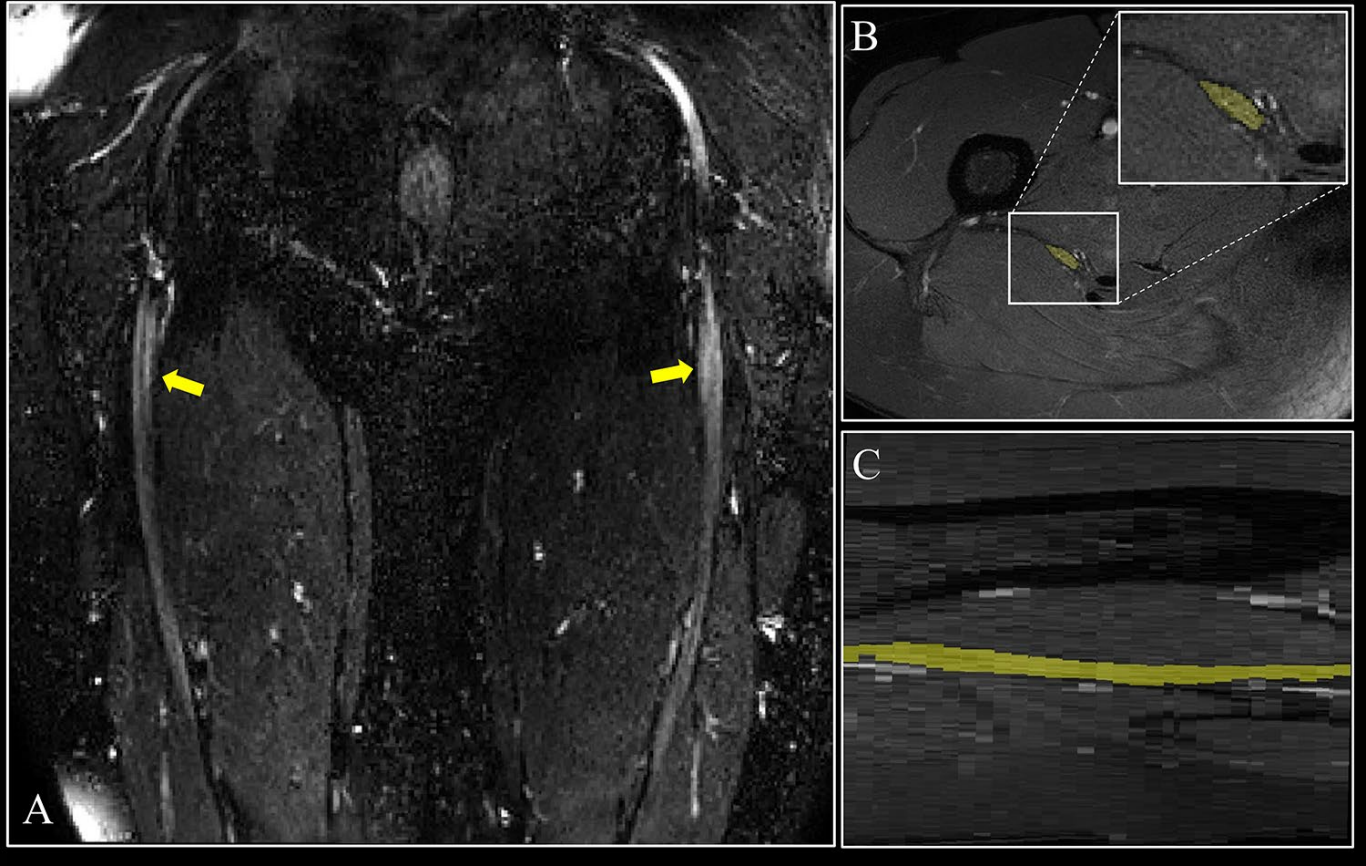
(**A**) Large field-of-view 3D SHINKEI image in the coronal plane showing the sciatic nerves (yellow arrows) in a healthy control; (**B**) Manual segmentation of the sciatic nerve slice-by-slice in the axial plane using the fat-suppressed T2-weighted acquisition (binary mask shown in yellow); (**C**) Segmentation of the sciatic nerve shown in the sagittal plane (binary mask shown in yellow).

The sciatic nerve was segmented manually in FSLview (http://www.fmrib.ox.ac.uk/fsl/) on the fat-suppressed T2-weighted images (Fig. 1), with separate binary masks created for each slice. The masks were created conservatively in order to ensure that only the sciatic nerve was included and not its border or any blood vessels running along or traversing the sciatic nerve. The high-resolution fat-supressed T2-weighted images and binary segmentation masks were subsequently resampled to the same resolution as the qMRI data and co-registered to the qMRI data with the use of reg_resample in NiftyReg (http://cmictig.cs.ucl.ac.uk/wiki/index.php/Reg_resample). Segmentation masks were visually inspected for correct alignment with the qMRI data (i.e., b = 0) and slightly adjusted if necessary, mostly downsized, to avoid partial volume artifacts^9^.

#### Pre-processing of qMRI data

All acquired qMRI volumes (i.e., DWI, qMT, IR) were pre-processed collectively using a previously reported denoising approach for dealing with multi-parametric data obtained with unified signal readout, and where the benefits of denoising were demonstrated specifically using a similar acquisition scheme^45^. The diffusion-weighted images were additionally motion corrected using slice-wise linear registration implemented in FSL (http://www.fmrib.ox.ac.uk/fsl), with registration transformations estimated among non-DWI images (i.e., b = 0), interleaved throughout the diffusion acquisition scheme as previously described^48^.

#### qMRI processing

- The DWI signals were fitted to the acquired multi-shell DW data using the DiPy Dkifit command (https://dipy.org/documentation/1.0.0./examples_built/reconst_dki/)^49,50^; from the fitting, standard DTI metrics were obtained, namely AD, RD, MD, FA, and also metrics derived from DKI^41^, namely AK, RK and MK.
- qMT data were analysed using a simplified two-pool model, as previously described^51^, to obtain estimates of BPF and T2_B_.
- Quantitative longitudinal relaxation time (qT1) maps were obtained from the IR data, by fitting a mono-exponential recovery model as previously described^47^.

**Figure 2:**
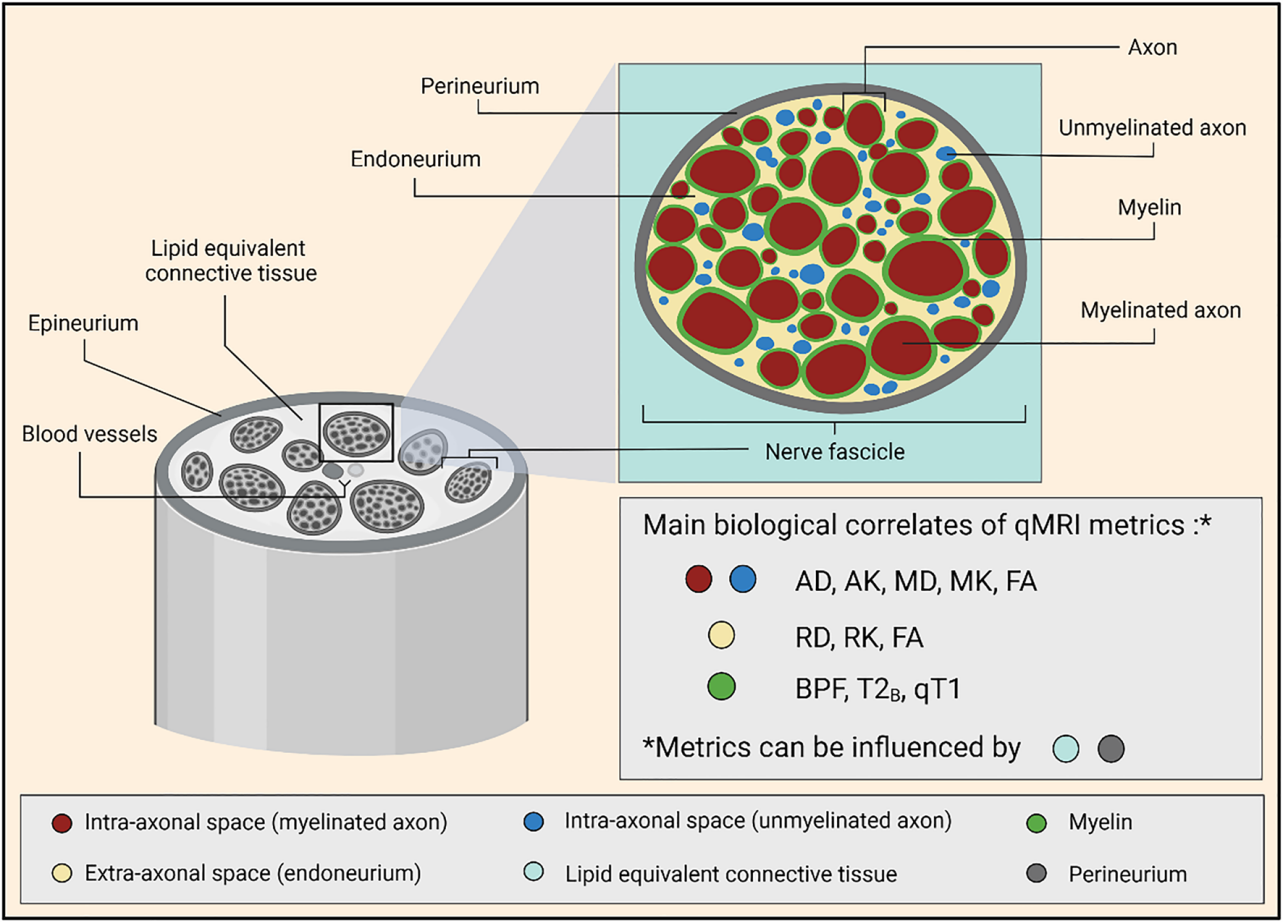
Schematic illustration of the healthy sciatic nerve cross-sectional anatomy at the level of the upper thigh, highlighting the main biological correlates of all qMRI metrics obtained in this study. In the healthy sciatic nerve, the main biological compartments underlying the qMRI metrics are myelin, intra-axonal water (in both myelinated and unmyelinated axons) and extra-axonal space (endoneurium). However, metrics can also be influenced by surrounding tissue compartments (e.g., perineurium, lipid equivalent connective tissue). **Note:** Although qT1 is known to be sensitive to myelin, the overall macroscopic T1 is rather unspecific, and is likely to be influenced by almost all biological compartments shown (e.g., myelin, amount of intra- and extra-axonal water, as well as potential exchange between the two water populations).

Figure 2 is a schematic illustration of the healthy sciatic nerve cross-sectional anatomy at the level of the upper thigh, highlighting the main biological correlates of the individual qMRI metrics obtained in this study.

### Reproducibility assessment

In order to assess scan-rescan reproducibility, six of the twelve subjects underwent a second scan on a separate visit (between 2 and 4 weeks from the first visit). In order to evaluate intra-rater reproducibility, the same rater (RB) segmented the images from the first visit of six participants twice on separate occasions, waiting at least two weeks between analyses. To examine inter-rater reproducibility, the data from the first visit of the six participants were analysed by a second rater (MY), with both raters working independently from one another. Then the scan-rescan reproducibility was assessed by a single rater (RB) conducting image segmentation on the data from the first and second visits.

### Statistical analysis

The mean and standard deviation (± SD) of all qMRI metrics across all study participants were calculated to report normative values in the sciatic nerve. In order to evaluate scan-rescan, intra- and inter-rater reproducibility, the percent coefficient of variation (%COV) was calculated using the mean and standard deviation from the repeated measures and the following equation: COV = [SD/mean] × 100%. Moreover, the intra-rater and inter-rater reproducibility were assessed using the intraclass correlation coefficient (ICC)^52^, which can be used to estimate the measurement error relative to the biological variability between subjects. In order to assess the intra- and inter-rater quality of the segmentations, the Dice similarity coefficient was calculated (DSC)^53^. The DSC is a measurement between two data sets calculated by dividing the size of the union of the two sets by the mean size of the two sets. The DSC range is from 0 to 1, where 0 implies no spatial overlap between two sets of binary segmentation masks and 1 represents total overlap.

## Results

**Figure 3:**
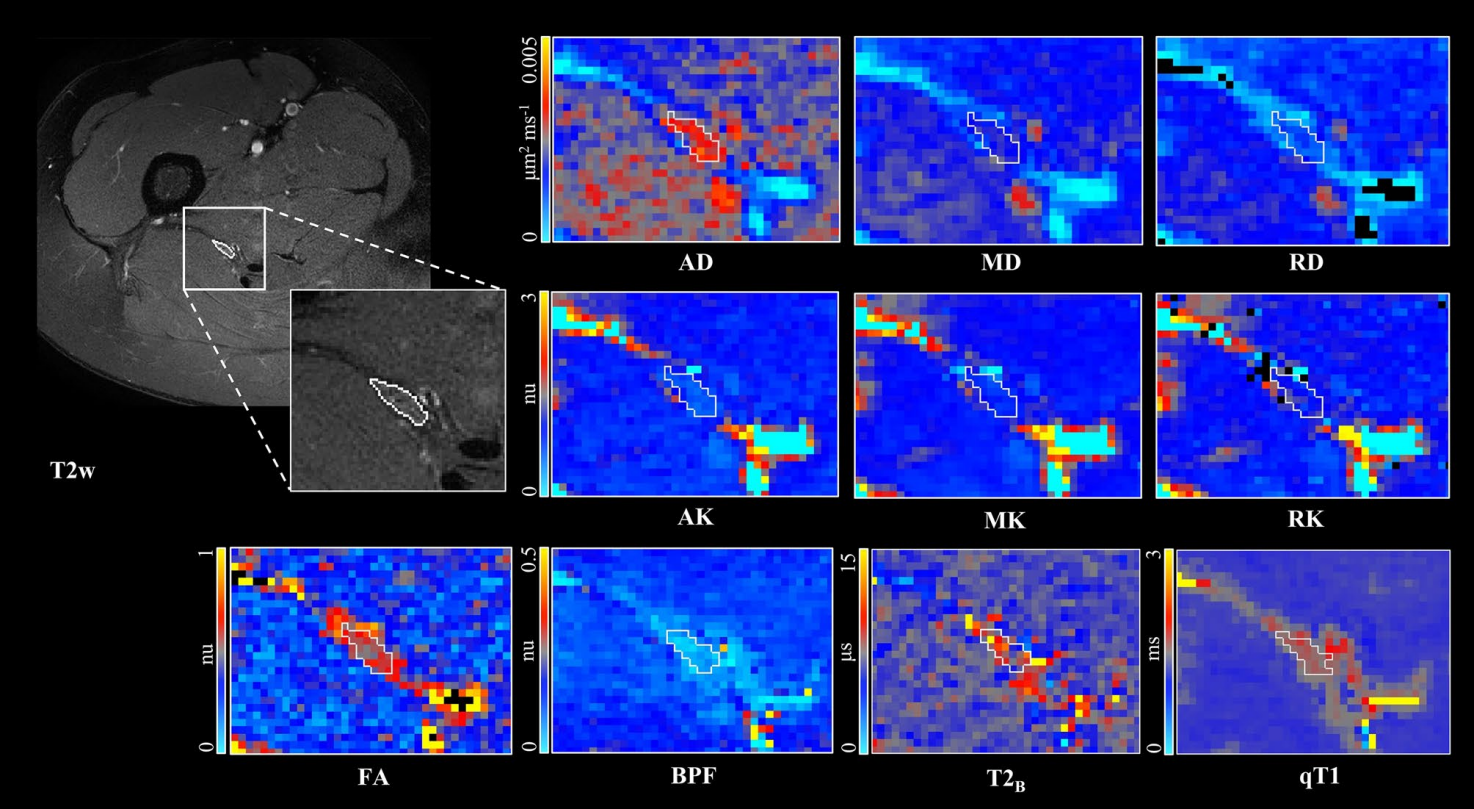
Example maps of standard diffusion tensor (DTI) derived metrics axial/radial/mean diffusivity (AD/RD/MD) and fractional anisotropy (FA), diffusion kurtosis imaging (DKI) metrics axial/radial/mean kurtosis (AK/RK/MK), quantitative magnetisation transfer (qMT) metrics bound pool fraction and bound pool transverse relaxation time (BPF/T2_B_) and quantitative longitudinal relaxation time (qT1), in the sciatic nerve of a healthy control.

**Figure 4:**
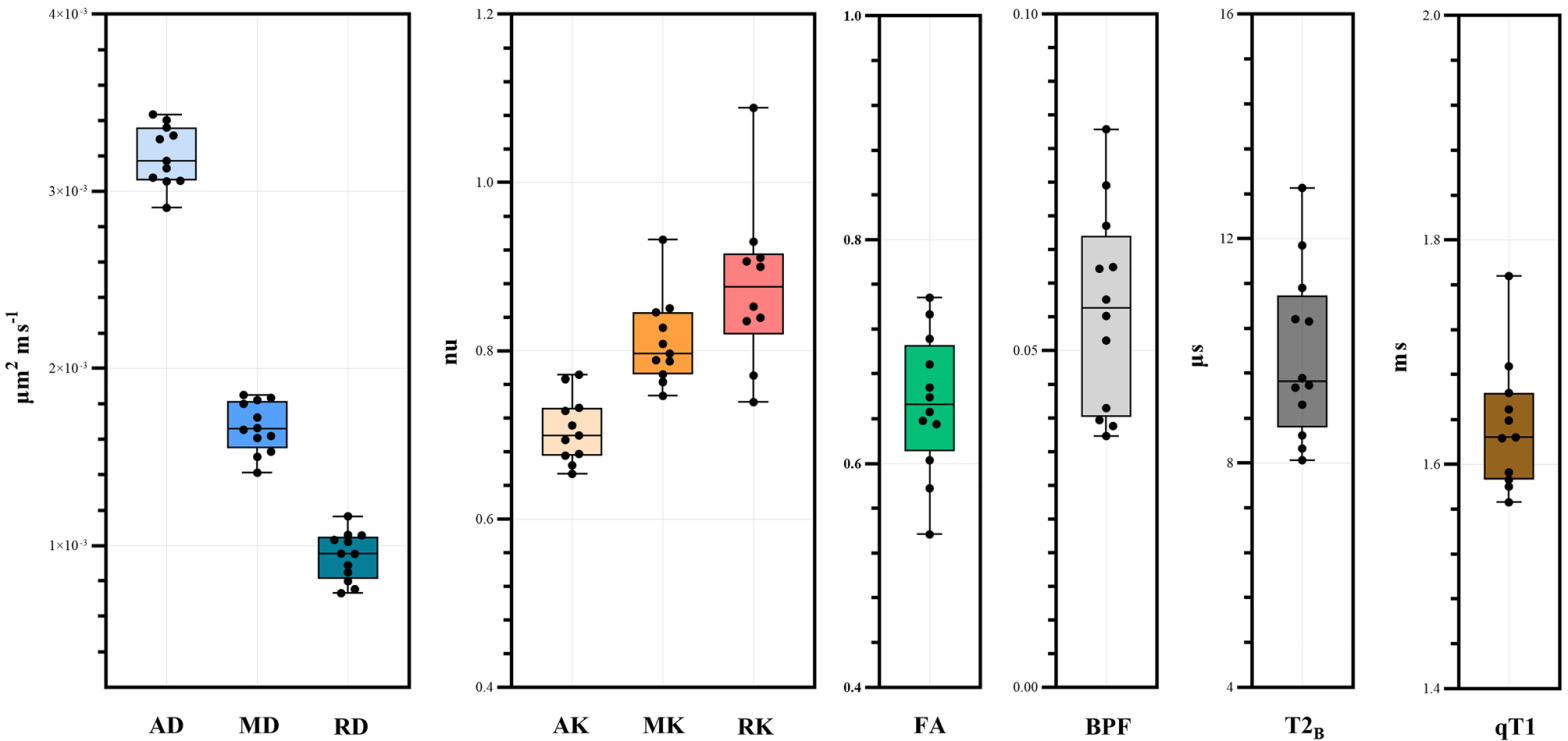
Boxplots with mean (± SD) values of the standard diffusion tensor (DTI) metrics axial/radial/mean diffusivity (AD/RD/MD) and fractional anisotropy (FA), diffusion kurtosis imaging (DKI) metrics axial/radial/mean kurtosis (AK/RK/MK), quantitative magnetisation transfer (qMT) metrics bound pool fraction and bound pool transverse relaxation time (BPF/T2_B_) and quantitative longitudinal relaxation time (qT1), in the sciatic nerve of 12 healthy volunteers.

Data from all 12 healthy volunteers, including data from repeated scanning in 6 healthy volunteers, were processed successfully without having to discard any because of motion-related artifacts. Figure 3 shows example maps of the standard DTI metrics (AD/RD/MD/FA), DKI metrics (AK/RK/MK), qMT (BPF/T2_B_) and IR (qT1) in a single healthy control. Table 1 shows the mean (± SD) values of all qMRI metrics calculated in all study participants (N = 12), with the corresponding boxplots of all metrics shown in Fig. 4.

**Table 1:** Mean (± SD) values of the standard diffusion tensor (DTI) metrics axial/radial/mean diffusivity (AD/RD/MD) and fractional anisotropy (FA), diffusion kurtosis imaging (DKI) metrics axial/radial/mean kurtosis (AK/RK/MK), quantitative magnetisation transfer (qMT) metrics bound pool fraction and bound pool transverse relaxation time (BPF/T2_B_) and quantitative longitudinal relaxation time (qT1), in the sciatic nerve of 12 healthy volunteers.

qMRI metrics	Mean (± SD)
AD, in μm^2^ ms^-1^	3.113 (0.001)
MD, in μm^2^ ms^-1^	1.670 (0.001)
RD, in μm^2^ ms^-1^	0.948 (0.001)
FA, *in nu*	0.649 (0.063)
AK, *in nu*	0.734 (0.088)
MK, *in nu*	0.844 (0.119)
RK, *in nu*	0.877 (0.186)
qT1, *in ms*	1635 (0.058)
BPF, *in nu*	0.054 (0.014)
T2_B_, in μs	9.975 (1.563)

### Reproducibility assessment

Table 2 shows the %COV results of the scan-rescan, intra-rater and inter-rater reproducibility assessments for each qMRI metric separately. In terms of scan-rescan reproducibility, all metrics had a COV value lower than 9% except for BPF, RK and T2_B_ (13.2%, 15.9% and 20.9%, respectively). In terms of the intra-rater results, all metrics had a COV value lower than 6%. Inter-rater results were similar to the intra-rater results, except for RK (10.6%). Table 3 shows the results of the intra-rater and inter-rater reproducibility assessments, expressed as the ICC. In terms of the intra-rater reproducibility, all metrics had ICC values higher than 0.86 (range 0.86–0.99). In terms of inter-rater reproducibility, all metrics had ICC values higher than 0.75, except for RK (0.64), with the overall range between 0.64 and 0.99. Table 4 shows the DSC results of the intra-rater and inter-rater assessments of image segmentation quality, with intra-rater mean (± SD) DSC of 0.69 (0.06) and inter-rater of 0.72 (0.07).

**Table 2:** Percent coefficient of variation (%COV) results of the standard DTI metrics axial/radial/mean diffusivity (AD/RD/MD) and fractional anisotropy (FA), DKI metrics axial/radial/mean kurtosis (AK/RK/MK), quantitative magnetisation transfer (qMT) metrics bound pool fraction and bound pool transverse relaxation time (BPF/T2_B_) and quantitative longitudinal relaxation time (qT1), for scan-rescan, intra-rater and inter-rater reproducibility assessments in 6 healthy volunteers.

	Scan-rescan	Intra-rater	Inter-rater
AD	3.7	2.4	1.9
MD	4.6	2.1	2.3
RD	8.8	4.7	3.2
FA	5.0	2.9	1.1
AK	4.3	2.2	2.1
MK	6.0	1.6	4.3
RK	15.9	2.7	10.6
qT1	2.8	1.4	0.9
BPF	13.2	5.2	5.6
T2_B_	20.9	4.6	6.1

**Table 3:** Intraclass correlation coefficient (ICC) results of the standard DTI metrics axial/radial/mean diffusivity (AD/RD/MD) and fractional anisotropy (FA), DKI metrics axial/radial/mean kurtosis (AK/RK/MK), quantitative magnetisation transfer (qMT) metrics bound pool fraction and bound pool transverse relaxation time (BPF/T2_B_) and quantitative longitudinal relaxation time (qT1), for intra-rater and inter-rater reproducibility assessments in 6 healthy volunteers.

	Intra-rater	Inter-rater
AD	0.96	0.83
MD	0.91	0.92
RD	0.93	0.97
FA	0.95	0.99
AK	0.97	0.89
MK	0.99	0.75
RK	0.95	0.64
qT1	0.86	0.99
BPF	0.98	0.96
T2_B_	0.91	0.86

**Table 4:** Dice similarity coefficient (DSC) results of the intra-rater and inter-rater image segmentation quality assessments in 6 healthy volunteers.

Subject	Intra-rater	Inter-rater
1	0.64	0.77
2	0.75	0.75
3	0.73	0.65
4	0.59	0.79
5	0.71	0.72
6	0.71	0.62
Mean	0.69	0.72
SD	0.06	0.07

## Discussion

In this pilot study, the feasibility of using advanced qMRI methods in conjunction with MRN in the PNS was investigated, with application to the sciatic nerve in healthy volunteers. The rationale for this study was based on the limited number of prospective in vivo studies employing advanced qMRI methods to study the PNS, despite their demonstrated ability to provide biophysically meaningful information pertinent to the underlying pathophysiological mechanisms involved in neurological disease^6^. The main reasons behind the limited implementation of qMRI methods in this context are likely to be related to the technical challenges associated with imaging the peripheral nerves, which include among others the small structure of the peripheral nerves, the surrounding tissue types with different magnetic susceptibility properties, the blood vessel distribution and flow effects, and RF coil and pulse sequence designs.

In this study, the reproducibility and reliability of a multi-parametric qMRI protocol was investigated by taking into account the main technical challenges involved to perform multi-shell DWI (for the estimation of DTI and DKI metrics), qMT (for the estimation of BPF and T2_B_) and IR (for the estimation of qT1) on a commercial 3T MRI system. A key feature of the qMRI protocol in this study included the use of ZOOM EPI, which benefits from the combined use of fat saturation with inner volume excitation, allowing alias free images with reduced sensitivity to the susceptibility artifacts that commonly affect long EPI readout acquisitions^43,44^. In addition, the qMRI protocol was acquired with a unified MRI signal readout, which enabled the acquisition of a uniquely rich set of image contrasts with matched resolution, distortion and intrinsic geometric alignment, all important aspects for successful multimodal characterisation of neural tissue microstructure^45^. Indeed, our unified-ZOOM EPI acquisition strategy is one of the main innovations of this work. The strategy makes high-quality multi-parametric qMRI feasible in the PNS, and has the potential of bringing advanced MRI methods for quantitative microstructural assessment one step closer to the clinic. Without the use of ZOOM-EPI for all our qMRI metrics, taking advantage of acceleration methods such as multi-slice excitation and parallel imaging reconstruction, this protocol would be much longer, and as a consequence a reduced choice of metrics would be sampled.

Previous studies have used DWI to examine the median, ulnar, radial, tibial and sciatic nerves, demonstrating the reliability of these measurements^9^, and how these maybe influenced by the anatomical location, age, sex, height, weight, body mass index (BMI)^10,11^, and by the nature of the pathological conditions implicating the PNS^12–16^. These studies have focussed on conventional DTI metrics (i.e., MD, RD, AD and FA), thus providing more specific information related to axon and myelin sheath integrity, typically invisible with conventional structural imaging.

This study sought to examine the feasibility of extending such previous approaches focussing on conventional DTI metrics by accounting for non-Gaussian diffusion through DKI^18^, in order to obtain additional information related to neural tissue microstructure. However, the additional information likely to be obtained through the use of DKI in the peripheral nerves may not be straightforward to interpret, given differences in tissue composition and microstructural organisation, as compared to the CNS. For example, differences in magnetic susceptibility properties between tissue types have also been shown to influence DKI metrics in the CNS^54^, and for this reason, similar investigations in the PNS are warranted to understand the possible source of image contrast in DKI. In terms of the standard DTI metrics, the mean values obtained in this study seem to follow a similar trend and appear to be in agreement with the results of previous studies examining the sciatic nerve in healthy controls^9,11^, although differences in technical and demographic factors do not permit a direct comparison. The potential value and feasibility of DKI measurements in the PNS has previously been explored in animal models^55^, but has only recently been addressed in vivo^56^. Therefore, more studies will be required in the future to understand the additional information related to tissue composition and microstructure that can potentially be obtained in pathological conditions affecting the PNS.

The role of qMT in the study of the PNS currently remains unexplored, despite the potential benefits previously demonstrated^32–37^, with most of the studies in the PNS currently relying on semi-quantitative assessments of myelin content through the use of MTR^24–28^. Future studies will therefore aim to clarify the potential benefits of using qMT in the PNS over semi-quantitative approaches like MTR. However, similar to MTR measurements, it is important to recognise the differences in tissue structure and composition of the nerves in the PNS as compared to the CNS, in order to interpret the origin of the qMT contrast in future investigations of pathological conditions affecting the PNS. In particular, assuming a two-pool description of biological tissues, the bound pool fraction in the PNS is represented by various tissue types, such as collagen, myelin and the proteins contained in the axons and Schwan cells^24,26^, thus dissimilar to the CNS tissue composition. The relative contribution of each tissue type to the qMT measurements remains unknown, and future research will be directed at addressing this gap.

The measurement of T1 relaxation time has provided invaluable information in a variety of clinical applications over the years^38^, although the limited implementation of T1 relaxometry in the study of the PNS is likely explained by the technical challenges associated with obtaining accurate T1 measurements^41^. Despite the availability of a variety of time-efficient T1 mapping methods to study the CNS^57–60^, their translation to the PNS may not be straightforward, and could be hampered by a number of site-specific factors such as radiofrequency pulse imperfections and incomplete magnetisation spoiling^41^. In this study, an IR-based T1 mapping method was used, benefiting from the aforementioned inherent qualities of ZOOM EPI acquisitions^43,44^, while making use of a slice shuffling scheme to allow T1 mapping of a large section of the sciatic nerve in a clinically acceptable scan time^47^. The mean T1 relaxation time in the healthy sciatic nerve was found to be longer (1635 ms) than previously measured in the healthy cervical spinal cord (1142 ms) using the same approach^47^, and also slightly longer than the previously reported T1 relaxation time in the healthy median nerve (1410 ms) at 3 T, using a different T1 mapping approach^61^. As previously mentioned, these variations might be explained by technical factors, anatomical location i.e., differences in tissue organisation and properties, and also demographic factors. More research will be required in the future to determine the role of T1 relaxometry in the study of the PNS.

In this study, reproducibility of the qMRI measurements was assessed by means of calculating the scan-rescan, intra-rater and inter-rater %COV from repeated measurements in a subset of study participants. Furthermore, intra-rater and inter-rater reproducibility was assessed by means of calculating the ICC. In order to assess the intra-rater and inter-rater quality of the image segmentations, the DSC was also calculated. Similar studies in the literature utilising a multi-parametric qMRI protocol with which to directly compare the reproducibility results of this study are not available. When comparing the %COV values obtained in this study with previous similar investigations in the brain and spinal cord, however, one must take into consideration the smaller size of the structure evaluated in this study. In particular, partial volume averaging is expected to have higher influence when assessing smaller structures, which is also supported by the moderate intra-rater and inter-rater DSC results in this study. Nevertheless, the ICC results of this study for the standard DTI metrics have demonstrated good to excellent agreement^62^, and are in line with previous similar investigations in the sciatic nerve^9^.

Finally, we acknowledge a number of limitations of our approach. One of main limitations of this study is that with 12 subjects the effect of age, gender, BMI, height and weight on the multi-parametric qMRI measurements obtained in this pilot study was not examined specifically, even though some of the demographic factors have been shown to influence various qMRI measurements significantly^11^. In addition, some of the unique technical features employed in this study, for example the use of ZOOM EPI, together with modifications to the sequence design in order to allow time-efficient acquisition of the qMRI metrics reported herein, may not be readily available in non-specialist centres, thus limiting the widespread implementation of the proposed qMRI protocol. Also, while DTI fitting is now part of most scanner software packages, analysis of advanced features such as DKI, qMT and qT1 are bespoke for our protocol.

In conclusion, this pilot study demonstrates the feasibility of combining MRN with a rich multi-parametric qMRI protocol based on a unified ZOOM-EPI readout, which enables the measurement of diffusion, quantitative magnetisation transfer and T1 relaxation properties of the healthy sciatic nerve in vivo. The reproducibility of the qMRI methods employed were found to be consistent with previous studies, demonstrated by the comparably low %COV, high ICC and DSC values obtained from scan-rescan sessions, intra-rater and inter-rater assessments. Future investigations involving a larger sample population will be required to confirm the findings of this study, to explore the demographic determinants of the qMRI measurements investigated, and to determine their potential role in pathological conditions implicating the PNS.

## Data Availability

The datasets used and analysed during the current study, as well as the code employed for the pre-processing and processing of the qMRI data, can be made available by the corresponding author on reasonable request.
